# Socio‐Epidemiological Correlates of Anemia Among Non‐Pregnant Females of East and West Uttar Pradesh, India: A NFHS‐5 Secondary Data Analysis Highlighting the Need for Precision Public Policy

**DOI:** 10.1002/puh2.70102

**Published:** 2025-08-18

**Authors:** Sonam Maheshwari, Richa Sinha, Neil Patel, Okashah Kaiwan, Avisham Goyal, Oroshay Kaiwan, Inderbir Padda, Nidhi Uniyal, Mohammed K. Suhail, Talha Bin Emran, Nirja Kaka, Yashendra Sethi

**Affiliations:** ^1^ Government Doon Medical College H.N.B. Medical Education University Dehradun Uttarakhand India; ^2^ PearResearch Dehradun India; ^3^ GMERS Medical College Himmatnagar Gujarat India; ^4^ University Hospitals Community Consortium Geauga Medical Center Chardon Ohio USA; ^5^ Department of Respiratory Medicine Graphic Era Institute of Medical Sciences Dehradun India; ^6^ Department of Medicine Metro Health Case Western Reserve University Cleveland Ohio USA; ^7^ Richmond University Medical Centre Staten Island New York USA; ^8^ Department of Medicine Graphic Era Institute of Medical Sciences Dehradun India; ^9^ Department of Public Health & Community Medicine IMU University Kuala Lumpur Malaysia; ^10^ Department of Pharmacy BGC Trust University Bangladesh Chittagong Bangladesh; ^11^ Department of Pharmacy Faculty of Allied Health Sciences Daffodil International University Dhaka Bangladesh; ^12^ Department of Medicine Subharti Medical College Swami Vivekanand Subharti University Meerut India

**Keywords:** anemia, anemia in India, anemia in non‐pregnant females, anemia in women, demographic correlates, East versus West Uttar Pradesh (UP), National Family Health Survey (NFHS), regional variations in UP, Uttar Pradesh

## Abstract

Anemia remains a persistent public health challenge in India, disproportionately affecting women and undermining maternal and reproductive health outcomes. Despite nationwide efforts, the burden of anemia continues to vary widely across regions, reflecting complex socio‐demographic determinants. This study aims to identify region‐specific risk factors for anemia among non‐pregnant women in East and West Uttar Pradesh (UP), India, using data from the National Family Health Survey (NFHS‐5). A secondary data analysis was conducted to explore socio‐demographic correlates of anemia among non‐pregnant females in East and West UP. Binary and ordinal logistic regression models were employed to analyze various explanatory variables. For variables not meeting the proportional odds assumptions, ordinal variables were binary categorized. Significant regional differences in the correlates of anemia were identified. In West UP, higher odds of anemia were associated with rural residency, alcoholism (odds ratio [OR] = 1.153; *p* < 0.01), heart disease (OR = 1.155; *p* < 0.01), younger age, economic disadvantage, illiteracy (OR = 1.335; *p* < 0.01), and underweight status (OR = 1.523; *p* = 0.012). In East UP, higher odds of anemia were associated with illiteracy (OR = 1.095; *p* = 0.010), having a child aged ≤48 months (OR = 1.296; *p* < 0.01), breastfeeding (OR = 1.067; *p* < 0.01), alcoholism (OR = 1.155; *p* < 0.01), hypertension (OR = 1.502; *p* < 0.01), and underweight status (OR = 1.523; *p* = 0.012). The study highlights significant differences in the socio‐epidemiological correlates of anemia between East and West UP. A universal public health policy is insufficient to address these disparities. Instead, a “Precision Public Policy” tailored to the specific needs of each region is necessary to improve implementation and outcomes.

## Graphical abstract

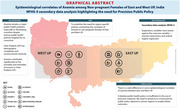

## 1 Introduction

Anemia is a medical condition characterized by a deficiency of healthy red blood cells or hemoglobin levels below 10.5 g/dL [1]. Anemia is a well‐known public health menace globally, affecting approximately one‐third of all adults and nearly two billion people worldwide [2]. The impact of anemia goes way beyond the impairment in quality of life, physical activity, and occupational life [3]. The widespread impact of anemia poses a considerable challenge to public health systems globally. Pregnant women are particularly susceptible to anemia, experiencing even higher prevalence rates [4, 5, 6]. According to estimates from the World Health Organization (WHO), in 2019, 40% of children aged 6–59 months, 37% of pregnant women, and 30% of women aged 15–49 years worldwide were affected by anemia [4]. This condition gives rise to various adverse health outcomes, including fatigue, impaired cognitive function, reduced work productivity, and an increased vulnerability to infections. Moreover, it significantly impedes the quality of life and socioeconomic development of communities [5].

Developing nations bear a substantial burden of anemia [5]. India, as the world's second‐most populous country, bears a notably high prevalence of anemia among developing nations, leading to the largest concentration of anemia cases on a global scale [5]. India's large population, diverse dietary patterns, and limited healthcare accessibility contribute to the persistent challenge of anemia in the country [7]. Within India, Uttar Pradesh (UP) stands out due to its massive population size and distinct regional characteristics. UP, the most populous state in northern India, is crucial when analyzing anemia prevalence due to its demographic complexity and socioeconomic diversity [8]. Had it been a country, it would have been the fifth most populous in the world, with a population even greater than Brazil [9]. The large population size and packed density in this region give rise to this conundrum. UP ranks fourth in terms of size, after only Rajasthan, Madhya Pradesh, and Maharashtra, as its landmass covers 243,286 square kilometers [10]. It can be broadly divided into East and West UP. UP West is geographically closer to Uttarakhand and the mountain regions of the Shivalik belt of the Himalayas, whereas UP East is closer to the state of Bihar [11]. The regions share geographical and topological similarities but vary largely in culture, food habits, customs, profession/source of income, population distribution, and endemic disease patterns. Given that the eastern half of the state has a per capita GDP that is almost exactly double that of the western half, the gaps in lives are staggering [12].

These regional disparities underscore the need for a comprehensive understanding of anemia prevalence and its impacts within UP [13].

The primary objective of this study is to shed light on the prevalence of anemia, its impact on India, and the regional variations within UP. The study aims to achieve the following objectives: (1) evaluate the prevalence of anemia in non‐pregnant married females of UP, with a specific focus on distinguishing between the eastern and western regions, and (2) investigate the socioeconomic factors and dietary contributors that contribute to anemia in this subgroup.

## 2 Materials and Methods

### 2.1 Study Setting and Data Sources

The study was performed on secondary data derived from the public database of the National Family Health Survey (NFHS‐5) surveys [14]. The NFHS is a large‐scale, multi‐phase survey that provides representative data on Indian families. It is similar to the Demographic and Health Surveys (DHS) conducted in various countries worldwide. The NFHS‐5, the fifth cycle of the survey, gathered data from 636,699 households across India. This iteration, covering the years 2019–21, offers detailed demographic, health, and nutrition information for each state and union territory in India. The NFHS is a comprehensive survey managed by a collaborative effort between the International Institute for Population Sciences (IIPS) in Mumbai, India; ICF in Calverton, Maryland, USA; and the East–West Center in Honolulu, Hawaii, USA. The Ministry of Health and Family Welfare (MOHFW) of the Government of India has appointed IIPS, Mumbai, as the lead agency for conducting NFHS‐5. The primary goal of each NFHS round is to provide essential data on health and family welfare indicators and to address emerging public health concerns.

NFHS employs a two‐stage stratified random sampling strategy for the selection of primary sampling units (PSUs), which comprise villages in rural areas and Census Enumeration Blocks in urban areas. In the first stage, PSUs are selected with a probability proportional to population size, followed by the selection of an equal number of households from each chosen PSU through systematic random sampling in subsequent stages. To ensure comprehensive data collection, the survey team is mandated to make up to three visits to a household if the eligible study participant is unavailable during the initial visit.

The NFHS uses the computer‐assisted personal interviewing (CAPI) method and includes four survey schedules—household, woman's, man's, and biomarker—conducted in the local language. The woman's schedule collects data on a range of topics, including personal characteristics, marriage, fertility, children's immunizations, childcare, nutrition, contraception, reproductive health, sexual behavior, HIV/AIDS, and domestic violence. The biomarker schedule focuses on measuring blood pressure, height, weight, hemoglobin levels, and random blood glucose levels for women aged 15–49. Results from all measurements and tests, except HIV testing, are provided to respondents immediately, along with relevant informational brochures. Trained health investigators conduct these tests and explain the results to the respondents.

### 2.2 Data Extraction—Population and Variables

**FIGURE 1:**
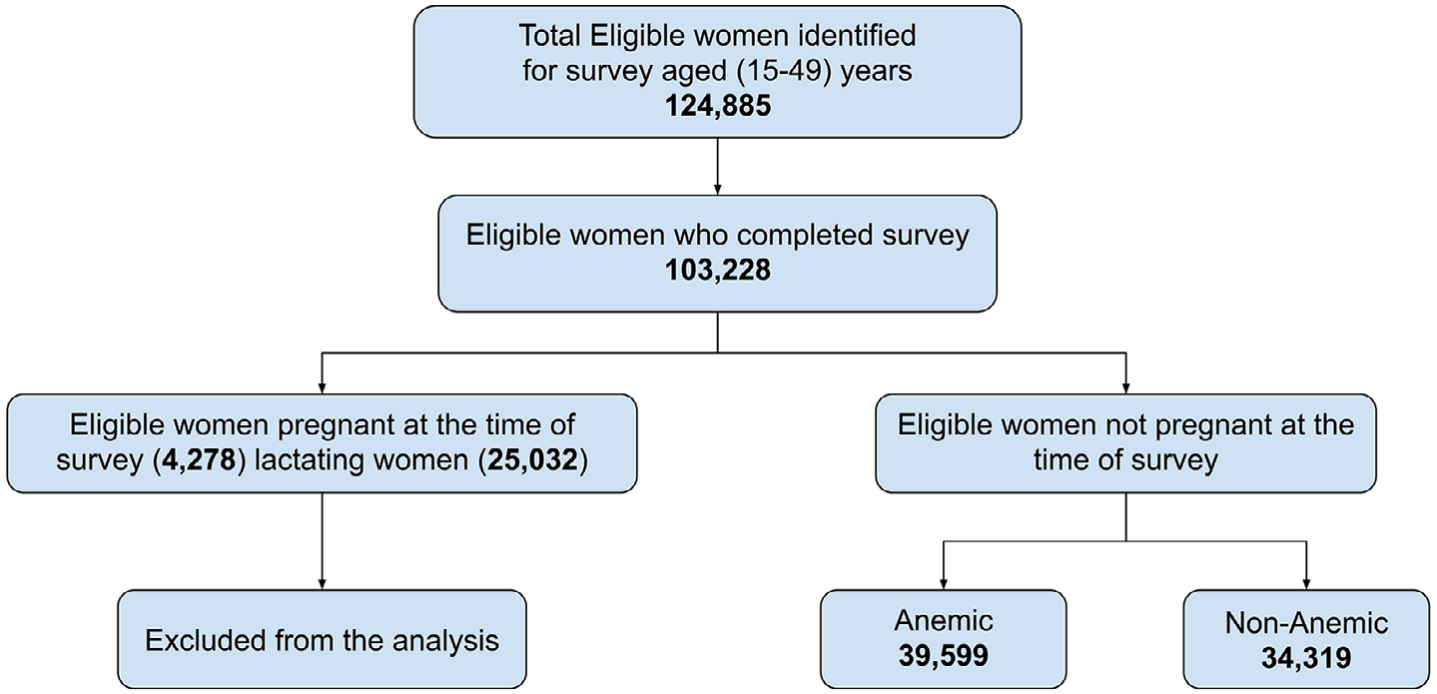
Flow diagram describing selection of the sample from dataset.

The data of interest were extracted for all major zones of UP (East, West, North, and South) and compared for East and West UP. This study considered the reproductive age group of non‐pregnant women—15–49 years in UP State. The selection of samples from the dataset was done as described in Figure 1. We excluded the women with missing data on Hb and other covariates, including place of residence, education level, household wealth, age at marriage, and so forth, in all rounds. Against the outcome variable of anemia (Hb), dependent variables were demographic, economic, and behavioral factors like age, children ever born, contraceptive use, place of residence, substance abuse, reproductive history, and current health status (Figure 1).

### 2.3 Case Definitions and Units of Measurement

The NFHS uses the following standards and definitions for data collection.

#### 2.3.1 Anemia

The NFHS classified anemia into three levels: mild (Hb level 10.0–11.9 g/dL), moderate (Hb level 7.0–9.9 g/dL), and severe (Hb level less than 7.0 g/dL). Any level of hemoglobin 11.9 g/dL or below was thus considered under the definition of anemia [14].

#### 2.3.2 Anthropometry

Height (in cm) and weight (in kg) were measured for women using a Seca 213 stadiometer and a Seca 874 digital scale, respectively. The body mass index (BMI) was calculated by dividing weight in kilograms by height in meters squared (kg/m^2^). BMI was then classified into the following categories: underweight (<18.5), normal weight (18.5–24.9), overweight (25.0–29.9), and obese (≥30.0).

#### 2.3.3 Hemoglobin Testing

Hemoglobin testing was conducted by health investigators with the consent of eligible women aged 15–49. Blood specimens were collected using a finger prick technique and gathered in a microcuvette. These samples were analyzed on‐site with a battery‐operated HemoCue Hb 201+ analyzer. Women identified with severe anemia (hemoglobin level < 7 g/dL) were referred to a health facility for further evaluation and treatment.

#### 2.3.4 Wealth Index

The wealth index is a measure of a household's living standards and reflects its relative wealth. Households are categorized into quintiles, ranging from the poorest (Code 1) to the richest (Code 5). Each household asset is assigned a weight based on principal components analysis. These weights are standardized to a normal distribution with a mean of zero and a standard deviation of one. The standardized scores are used to determine breakpoints for the wealth quintiles: poorest (lowest), poorer (second), middle, richer (fourth), and richest (highest) [14].

### 2.4 Statistical Analysis

Data from the NFHS were retrieved and analyzed with IBM SPSS for Windows version 28.0. By visualizing the percentage point difference, the NFHS‐5 data were also evaluated for disparities in the distribution of the outcome prevalence by place of residence. Univariate and bivariate analyses were used to assess the level of anemia among reproductive age group women of the East and West zones of UP. In bivariate analysis, a chi‐square test and odds ratio (OR) were used to check the statistical significance of the differences in anemia prevalence across demographic, social, behavioral, and health characteristics. To identify the factors linked with anemia among women, we also applied multivariate techniques to study the relationship between the potential risk factors and the severity of anemia after checking the multicollinearity using VIF (variance inflation factor) values. Binary logistic regression was used by combining the groups of all anemia patients into one and comparing them with patients without anemia. As anemia status is an ordinal variable, ordinal logistic regression was employed. The proportional odds model for the categorical variable *Y* with *C* ordered categories and a set of *P* explanatory variables for the *i*th subject is described as follows:

*X_i_* = (*x_i_*_1_, *x*_2_ *_i_*, …..,*x_pi_*), *I* = 1,2,….*n* is given as

$$\begin{array}{ccc} & & logit\left(\mathrm{Pr}\left(Y_{i}\leq i|x_{i}\right)\right)=log\left(\frac{\pi (x_{i})}{1-\pi (x_{i})}\right)\\ & & \;=\;\alpha \_i-\beta \_1\;x\_i1-\beta \_p\;x\_\left(pl@\right)\\ & & \;=\;\alpha \_i-X\_i\;\beta \end{array}$$

where *I* is the intercept for the cumulative logit, *π*(*X_l_*) *= *Pr(*Y_l_ ≤ i*|*xl*), and *β* is a column vector of *P* regression coefficients.

## 3 Results

The Table 1 shows that the prevalence of anemia (severe, moderate, and mild combined) is slightly higher in rural areas compared to urban areas across both East and West UP. Mild anemia is the most common form in all regions, with around 40% prevalence, whereas severe anemia remains low, under 1.5% in all subgroups.

**TABLE 1:** Prevalence of anemia among females of East and West Uttar Pradesh (UP).

	West UP				East UP			
Anemia level	Urban		Rural		Urban		Rural	
	Sample	Percentage	Percentage	Percentage	Sample	Percentage	Sample	Percentage
Severe	161	1.1	293	1.4	46	0.9	290	1.0
Moderate	1712	11.8	2789	13.1	581	12.0	3530	12.1
Mild	5657	38.9	8530	40.1	2003	41.3	11,837	40.7
Normal	7007	48.2	9668	45.4	2222	45.8	13,434	46.2
Total	14,537	100.0	21,280	100.0	4852	100.0	29,091	100.0

### 3.1 Demographic Correlates of Anemia in Females of UP

Urban women in West UP had higher odds of severe anemia compared to their rural counterparts, with an OR of 1.075 (95% CI: 1.051–1.100). Women who had received a blood transfusion also had increased odds of severe anemia (OR = 1.128, 95% CI: 1.075–1.185). Additionally, hypertensive women in West UP had significantly higher odds of severe anemia (OR = 1.376, 95% CI: 1.344–1.409) (Tables 2 and 3).

**TABLE 2:** Demographic and socio‐epidemiological correlates of anemia among females in West Uttar Pradesh (UP).

Variables (chi‐square and *p* value)			Severely anemic		Moderately anemic		Mildly anemic		Non‐anemic		Crude odds ratio
Chi‐square	*p* value	Age									
102.60	0.000	15–19	90	1.1	1050	12.4	3443	40.6	3889	45.9	1.187(0.000) 1.200(0.000) 1.280(0.000) 1.099(0.036) 1.139(0.005) 1.108(0.031)
		20–24	125	1.6	1161	15.1	2897	37.7	3509	45.6	
		25–29	74	1.3	909	15.5	2298	39.2	2582	44.0	
		30–34	67	1.4	626	13.5	1720	37.2	2211	47.8	
		35–39	66	1.6	536	12.8	1624	38.7	1968	46.9	
		40–44	53	1.4	488	13.1	1415	37.9	1778	47.6	
		45–49	42	1.2	390	11.4	1268	37.2	1712	50.2	
**Type of residence**											
39.66	0.000	Urban	182	1.2	1931	12.6	5818	38.1	7356	48.1	0.894(0.000)
		Rural	335	1.5	3229	14.2	8847	39.0	10293	45.3	
**Education**											
151.10	0.000	No education	236	1.8	1986	14.9	5156	38.8	5926	44.5	1.335(0.000) 1.214(0.000) 1.249(0.000)
		Primary	75	1.5	702	14.1	1858	37.4	2328	46.9	
		Secondary	172	1.2	1955	13.4	5733	39.2	6747	46.2	
		Higher	34	0.7	517	10.1	1918	37.5	2648	51.7	
**Religion**											
0.71	0.871	Hindu	356	1.3	3607	13.6	10208	38.5	12347	46.6	0.986(0.532)
		Non‐hindu	161	1.4	1553	13.5	4457	38.8	5302	46.2	
**Wealth index**											
106.83	0.000	Poorest	82	1.8	655	14.5	1707	37.7	2084	46.0	1.151(0.000) 1.120(0.000) 1.243(0.000) 1.195(0.000)
		Poorer	115	1.6	1051	14.3	2756	37.5	3434	46.7	
		Medium	121	1.6	1109	14.3	3112	40.1	3426	44.1	
		Richer	117	1.4	1181	13.9	3368	39.6	3831	45.1	
		Richest	82	0.8	1164	11.8	3722	37.8	4874	49.5	
**Total children ever born**											
86.32	0.000	0	165	1.2	1667	11.8	5502	38.9	6817	48.2	0.904(0.000) 1.060(0.115)
		≥2	307	1.5	2914	14.4	7809	38.5	1568	44.2	
		<2	45	1.3	579	16.3	1354	38.2	9264	45.6	
**Sex of last child**											
0.463	0.927	Male	208	1.5	2063	14.6	5466	38.6	6424	45.4	1.196(0.000)
		Female	144	1.5	1430	14.8	3697	38.2	4408	45.5	
**Age of last child**											
274.33	0.000	≤48 months	170	1.7	1805	18.2	3962	40.0	3964	40.0	1.030(0.086)
		>48 months	182	1.3	1688	12.1	5201	37.3	6868	49.3	
**Age at marriage**											
17.03	0.009	<18 Years	191	1.6	1615	13.9	4365	37.7	5411	46.7	1.033(0.687) 1.099(0.239)
		18–26 years	199	1.4	2125	15.2	5432	38.2	6317	45.2	
		>26 Years	5	0.8	83	12.8	252	38.9	308	47.5	
**Currently breastfeeding**											
174.71	0.000	Yes	94	1.6	1025	17.0	2544	42.3	2349	39.1	1.090(0.000)
		No	423	1.3	4135	12.9	12121	37.9	15300	47.8	
**Smoking**											
1.18	0.759	Yes	512	1.4	5116	13.6	14,541	38.6	17,481	46.4	1.030(0.787)
		No	5	1.5	44	12.9	124	36.4	168	49.3	
**Drink alcohol**											
1.433	0.698	Yes	0	0	1	6.7	5	33.3	9	60.0	1.153(0.000)
		No	517	1.4	5159	13.6	14,660	38.6	17,640	46.5	
**Tobacco use**											
1.463	0.691	Yes	1	1.3	7	9.0	31	39.7	39	50.0	0.867(0.530)
		No	516	1.4	5153	13.6	14,634	38.6	17,610	46.4	
**Ever had a blood transfusion**											
31.41	0.000	Yes	50	2.2	366	16.2	869	38.5	970	43	1.159(0.001)
		No	467	1.3	4794	13.4	13,796	38.6	16,679	46.7	
**Diabetes**											
		Yes	4	1.1	41	11.1	120	32.5	204	55.3	0.698(0.001)
11.81	0.008	No	512	1.4	5111	13.6	14,497	38.7	17,369	46.3	
**Asthma**											
		Yes	3	1	33	11.3	99	33.9	157	53.8	0.744(0.102)
6.45	0.092	No	514	1.4	5125	13.6	14,562	38.6	17,481	46.4	
**Hypertension**											
701.85	0.000	Yes	88	0.7	1247	9.3	4727	35.4	7293	54.6	0.831(0.000)
		No	429	1.7	3904	15.9	9890	40.3	10,297	42.0	
**Heart disease**											
2.41	0.493	Yes	9	1.4	78	12.2	236	37.0	314	49.3	1.155(0.000)
		No	508	1.4	5082	13.6	14,426	38.6	17,327	45.4
**Body mass index (BMI)**											
		Thin	129	24.9	1058	20.5	3459	23.6	6458	36.5	0.124(0.000)
5153.89	0.000	Normal	279	53.9	2056	39.8	5417	36.9	9856	55.7	0.135(0.000)
		Obese	109	21.08	2046	39.6	5789	39.5	1365	7.7	

**TABLE 3:** Demographic and socio‐epidemiological correlates of anemia among females in East Uttar Pradesh (UP).

Variables (chi‐square and *p* value)			Severely anemic		Moderately anemic		Mildly anemic		Non‐anemic		Crude odds ratio
		Age									
Chi‐square	*p* value		Frequency	% Age	Frequency	% Age	Frequency	% Age	Frequency	% Age	
62.15	0.000	15–19	83	1.0	1040	12.4	3484	41.4	3806	45.2	1.061(0.150) 1.020(0.651) 1.007(0.867) 0.975(0.577) 0.978(0.641) 1.001(0.987)
		20–24	84	1.3	926	14.0	2555	38.5	3066	46.2	
		25–29	43	0.8	771	14.3	2071	38.4	2511	46.5	
		30–34	46	1.0	594	12.8	1800	38.8	2195	47.4	
		35–39	42	1.0	486	11.7	1661	40.0	1962	47.3	
		40–44	51	1.5	407	11.8	1383	40.0	1613	46.7	
		45–49	24	0.7	373	11.5	1333	41.1	1517	46.7	
	**Type of residence**										
1.83	0.609	Urban	48	1.0	636	12.6	2048	40.5	2319	45.9	1.023(0.453)
		Rural	325	1.1	3961	12.8	12,239	39.6	14,351	46.5	
	**Education**										
30.37	0.000	No education	176	1.3	1823	13.2	5451	39.5	6358	46.0	1.095(0.010) 1.097(0.035) 1.084(0.020)
		Primary	35	0.9	517	13.1	1581	40.0	1816	46.0	
		Secondary	139	1.0	1753	12.7	5548	40.1	6409	46.3	
		Higher	23	0.5	504	11.7	1707	39.5	2087	48.3	
	**Religion**										
1.07	0.784	Hindu	311	1.0	3872	12.8	12,062	39.9	14,009	46.3	1.024(0.404)
		Non‐hindu	62	1.1	725	12.8	2225	39.2	2661	46.9	
	**Wealth index**										
55.89	0.000	Poorest	157	1.3	1653	13.7	4880	40.4	5383	44.6	1.122(0.004) 1.019(0.638) 1.004(0.921) 0.983(0.708)
		Poorer	103	1.1	1248	13.2	3671	38.8	4449	47.0	
		Medium	49	0.8	790	12.2	2584	39.7	3078	47.3	
		Richer	39	0.8	533	11.5	1838	39.7	2214	47.9	
		Richest	25	0.6	373	0.8	1314	36.3	1546	39.2	
	**Total children ever born**										
18.71	0.005	0	135	1.0	1641	12.5	5153	39.3	6180	47.1	0.964(0.103) 1.068(0.075)
		≥2	38	1.1	521	14.9	1383	39.5	1563	44.6	
		<2	200	1.0	2435	12.6	7751	40.1	8927	46.2	
	**Sex of last child**										
12.13	0.007	Male	121	0.9	1663	12.5	5374	40.4	6153	46.2	1.192(0.000)
		Female	117	1.2	1293	13.6	3760	39.5	4337	45.6	
	**Age of last child**										
130.80	0.000	≤48 months	121	1.2	1489	15.2	4031	41.2	4136	42.3	1.296(0.000
		>48 months	117	0.9	1467	11.2	5103	39.1	6354	48.7	
	**Age at marriage**										
9.107	0.168	<18 Years	172	1.1	2008	12.7	6159	39.0	7446	47.2	0.898(0.260) 0.945(0.562)
		18–26 years	88	0.9	1278	13.7	3654	39.5	4273	45.9	
		>26 Years	4	0.9	61	13.4	187	41.2	202	44.5	
	**Currently breastfeeding**										
96.81	0.000	Yes	76	1.1	1019	14.3	3098	43.4	2941	41.2	1.067(0.000)
		No	297	1.0	3578	12.4	11,189	38.9	13,729	47.7	
	**Smoking**										
96.81	0.000	Yes	366	1.0	4548	12.8	14,134	39.9	16,404	46.3	0.786(0.000)
		No	7	1.5	49	10.3	153	32.2	266	56.0	
	**Drink alcohol**										
1.11	0.774	Yes	0	0.0	13	13.7	39	41.1	43	45.3	1.155(0.000)
		No	373	1.0	4584	12.8	14,248	39.8	16,627	46.4	
	**Tobacco use**										
1.730	0.630	Yes	2	1.7	14	12.2	51	44.3	48	41.7	1.209(0.316)
		No	371	1.0	4583	12.8	14236	39.8	16622	46.4	
	**Ever had a blood transfusion**										
13.95	0.003	Yes	18	1.6	174	15.8	420	38.3	486	44.3	1.159(0.001)
		No	355	1.0	4423	12.7	13867	39.8	16184	46.5	
	**Diabetes**										
10.48	0.015	Yes	18	1.6	174	15.8	420	38.3	486	44.3	0.794(0.025)
		No	355	1.0	4423	12.7	13867	39.8	16184	46.5	
	**Asthma**										
5.80	0.122	Yes	10	1.7	74	12.7	211	36.1	289	49.5	0.822(0.131)
		No	363	1.0	4523	12.8	14075	39.8	16378	46.3	
	**Hypertension**										
447.18	0.000	Yes	100	0.6	1622	10.1	5973	37.3	8309	51.9	1.502(0.000)
		No	264	1.4	2898	15.1	8031	41.7	8048	41.8	
	**Heart disease**										
4.72	0.193	Yes	6	1.1	65	12.0	194	35.9	275	50.9	0.832(0.034)
		No	367	1.0	4531	12.8	14093	39.8	16935	46.3	
	**Body mass index (BMI)**										
740.96	0.000	Thin	98	26.3	1958	42.6	5247	37.2	4528	26.3	1.523(0.012)
		Normal	189	50.7	2056	44.7	6258	44.4	9424	54.8	0.852(0.124)
		Obese	86	23.1	582	12.7	2782	19.7	3258	18.9	

### 3.2 Socioeconomic Factors and Dietary Correlates for Anemia

The odds of being anemia were higher among women in all age groups (15–19, 20–24, 25–29, 30–34, 35–39, and 40–44) than women in age group 45–49 in both West UP; however, the age of the women showed no significant association in East UP (Tables 3 and 4).

**TABLE 4:** Association between anemia and obesity.

		Urban						Rural					
	Anemia level	Thin		Normal		Obese		Thin		Normal		Obese	
Area		Sample	Percentage	Sample	Percentage	Sample	Percentage	Sample	Percentage	Sample	Percentage	Sample	Percentage
West UP	Severe	36	22.6	86	54.1	37	23.3	110	57.5	168	57.3	15	5.1
	Moderate	390	22.9	903	52.9	413	24.2	858	30.9	1605	57.7	318	11.4
	Mild	1047	18.6	3002	53.2	1595	28.3	2311	27.2	4911	57.7	1288	15.1
	Normal	1072	15.3	3616	51.7	2305	33.0	2273	23.6	5542	57.5	1288	15.1
	Total	2545	17.5	7607	52.5	4550	30.0	5552	26.2	12266	57.6	3452	16.3
East UP	Severe	13	28.3	32	69.6	1	2.2	114	39.4	159	55.0	16	5.5
	Moderate	161	27.7	306	52.7	114	19.6	1107	31.4	2091	59.3	331	9.4
	Mild	439	21.9	1060	52.9	503	25.1	3364	28.5	7072	59.8	1382	11.7
	Normal	404	18.2	1240	55.9	576	25.9	3524	26.2	7889	58.8	2013	15.0
	Total	1017	21.0	2638	54.4	1194	24.6	8109	27.9	17211	59.2	3742	12.9

Abbreviation: UP, Uttar Pradesh.

The odds of being anemic were higher among illiterate women (OR = 1.335) in West UP and (OR = 1.095) in East UP women compared to women with higher education (Tables 2 and 3). Women from quintiles in all groups based on income had higher odds of being anemic than women from the richest age group in West UP, whereas the data in East UP were insignificant. However, the women from the poorest group, even in East UP, had higher odds of being anemic compared to the richest group (OR = 1.122) (Tables 5 and 6). The binary logistic regression shows that the odds of being anemic were higher among women whose child is aged ≤48 months in East UP (OR = 1.335), whereas the data in West UP were insignificant (Tables 3 and 4). The odds of being anemic were lower in women having diabetes than those without in both East and West UP. The odds were higher in women having hypertension in East UP and lower in West UP compared to those without hypertension (Tables 2 and 3).

**TABLE 5:** Parameter estimates of related covariates in ordinal logistic regression model for West Uttar Pradesh (UP).

				95% confidence interval	
Variables	Category	Odds ratio	*p* value	LB	UB
Age	15–19	0.936	0.003	0.895	0.978
	20–24	0.867	0.000	0.829	0.907
	25–29	0.852	0.000	0.813	0.894
	30–34	0.923	0.002	0.878	0.970
	35–39	0.927	0.004	0.882	0.976
	40–44	0.932	0.008	0.884	0.981
Residence	Urban	1.075	0.000	1.051	1.100
Education	No education	0.805	0.000	0.777	0.835
	Primary	0.845	0.000	0.809	0.883
	Secondary	0.859	0.000	0.829	0.891
Wealth index	Poorest	0.886	0.000	0.852	0.922
	Poorer	0.901	0.000	0.871	0.932
	Middle	0.877	0.000	0.848	0.907
	Richer	0.898	0.000	0.868	0.927
Age at marriage	<18 Years	0.948	0.235	0.867	1.036
	18–26 years	0.911	0.040	0.834	0.996
Currently breastfeeding	Yes	1.205	0.000	1.168	1.244
Smoking	Yes	0.957	0.468	0.849	1.078
Drink alcohol	Yes	0.691	0.723	0.395	1.211
Tobacco use	Yes	0.862	0.240	0.673	1.104
Ever had a blood transfusion	Yes	1.128	0.000	1.075	1.185
Diabetes	Yes	0.845	0.004	0.754	0.947
Thyroid	Yes	0.940	0.168	0.861	1.206
Hypertension	Yes	1.376	0.000	1.344	1.409
Heart disease	Yes	0.910	0.100	0.813	1.018
Body mass index	Thin	0.874	0.004	0.478	0.981
	Normal	0.739	0.000	0.678	0.902

**TABLE 6:** Parameter estimates of related covariates in ordinal logistic regression model for East Uttar Pradesh (UP).

				95% confidence interval	
Variables	Category	Odds ratio	*p* value	LB	UB
Age	15–19	1.559	0.039	1.437	1.692
	20–24	0.905	0.000	0.823	0.995
	25–29	0.833	0.001	0.756	0.918
	30–34	0.841	0.355	0.760	0.931
	35–39	0.952	0.951	0.857	1.057
	40–44	0.997	0.563	0.899	1.106
Residence	Urban	1.001	0.927	0.969	1.035
Education	No education	0.925	0.000	0.890	0.961
	Primary	0.935	0.006	0.891	0.981
	Secondary	0.945	0.004	0.909	0.981
Religion	Hindu	0.994	0.732	0.963	1.027
Wealth index	Poorest	0.908	0.000	0.870	0.949
	Poorer	0.948	0.020	0.907	0.992
	Middle	0.983	0.471	0.938	1.030
	Richer	1.001	0.961	0.952	1.052
Age at marriage	<18 years	1.042	0.448	0.938	1.157
	18–26 years	1.007	0.897	0.906	1.120
Currently breastfeeding	Yes	1.119	0.000	1.087	1.152
Smoking	Yes	0.849	0.001	0.767	0.938
Drink alcohol	Yes	1.009	0.937	0.805	1.266
Tobacco use	Yes	0.755	0.241	0.506	1.187
Ever had a blood transfusion	Yes	1.127	0.014	1.024	1.240
Diabetes	Yes	1.022	0.840	0.830	1.259
Thyroid	Yes	0.866	0.106	0.728	1.031
Hypertension	Yes	1.354	0.000	1.287	1.425
Heart disease	Yes	0.841	0.355	0.760	0.931
Body mass index	Thin	1.259	0.018	0.958	1.359
	Normal	0.869	0.000	0.784	0.933

We found that severe anemia was more prevalent among obese in urban areas of UP, especially West UP. However, in urban areas, 23.3% of obese individuals had severe anemia, whereas in rural areas, only 5.1% were obese with severe anemia (Table 4). Furthermore, in West UP, the odds of anemia were significantly higher among women with alcohol dependency (OR = 1.153, *p* < 0.01), heart disease (OR = 1.155, *p* < 0.01), younger age (e.g., ORs for ages 15–19 and 20–24 years), economic vulnerability, or those with lower educational levels. However, being underweight (OR = 0.874, *p* = 0.004) appeared to be associated with a lower risk of anemia in this region. In Eastern UP, the odds of anemia were elevated among women who were currently breastfeeding (OR = 1.119, *p* < 0.01), had hypertension (OR = 1.354, *p* < 0.01), or were underweight (OR = 1.259, *p* = 0.018). Contrary to expectations, illiteracy (OR = 0.925, *p* = 0.000) appeared to be associated with a lower risk of anemia (Tables 4, 5, 6).

## 4 Discussion

The study was conducted to evaluate the prevalence of anemia in non‐pregnant females of eastern and western regions of UP, India, and investigate the socioeconomic factors and dietary contributors that contribute to the same. The authors found that there were significant differences between the regions.

The odds of being anemic for urban women residents were almost 11% lesser than those living in rural areas in West UP, which is in conjunction with the reported literature of rural setups having higher risk of anemia than urban areas. This disproportionality is explained through difficulties in securing balanced diets, poor living conditions, poverty, lack of access to healthcare services, and ignorance/poor literacy rates [15, 16, 17, 18, 19, 20, 21, 22, 23]. Further, the odds of being anemic were higher among women in younger age groups (<45 years of age) than women in age group 45–49 in West UP; however, the age of the women showed no significant association in East UP. Moreover, the odds of being anemic were 33.5% higher among illiterate women in West UP and 9.5% higher in East UP women compared to women with higher education (Tables 2, 3, 4). This can be explained by the lack of proper health information, unhealthy menstrual practices, and altered economics [19, 23, 24].

Interestingly, the women from all income‐based quintiles had higher odds of being anemic than richest group in West UP, but interestingly, only the women from the poorest group, in East UP, had higher odds of being anemic compared to the richest group. The authors opine that this is a significant finding highlighting the difference for middle class population in both regions that is often overlooked (Tables 5 and 6). East UP has a more apparent economic disparity contributing to stark difference between rich and poor, whereas maintaining the basic nutritional and economic needs for people belonging to the middle class [18, 19, 20, 21, 22, 23, 24].

It is worth noting that the women who had a blood transfusion were 12.8% more likely to be severely anemic, indicating a continuous and persisting burden of anemia despite existing public health interventions (Tables 2 and 3). The odds of being anemic were lower in women having diabetes than those without in both East and West UP. The odds were higher in women having hypertension in East UP and lower in West UP compared to those without hypertension (Tables 2 and 3). This is in contrast to the expected higher association of anemia with DM, demonstrated by Mistry et al. and Soliman et al. [24, 25]. The association of anemia with these co‐morbidities forms an interesting association for the region and requires further exploration before reaching plausible conclusions to plan an intervention. From what we can best understand, the existence of a treatment plan for diabetes asserts a stricter control of diet and lifestyle that might have positively influenced the results. The authors understand that the existence of a treatment plan catering to the co‐morbidities might assert a stricter diet and lifestyle control that might have positively influenced the results. On the other hand, the study found varying effects of hypertension on anemia across the two regions. In East UP, women with hypertension had higher odds of anemia, whereas in West UP, the odds were lower. This variance might be attributed to regional differences in healthcare access and management of hypertension. The data suggest that although diabetes management may mitigate anemia risk, the relationship between hypertension and anemia might be influenced by local healthcare practices and regional health disparities. Further research is needed to explore these complex interactions and develop region‐specific interventions.

The responsible recommendation from the association can just be an overt benefit from shift to a healthier lifestyle [24]. Lastly, we observed that odds of having anemia were higher in both regions for alcoholics, which was expected per the current evidence as alcohol can lead to acute hemolysis, nutritional anemia, sideroblastic anemia, and cirrhosis [25, 26, 27, 28]. Ethanol is also known for its inhibitory effect on heme synthesis, anti‐folate activity, and direct hematotoxicity [20, 21, 22].

An association between BMI and anemia has been proposed earlier as well [29]. It has been observed in many studies that obese women have significantly lower serum iron levels and higher soluble transferrin receptor levels. Further, the fat mass has been implicated as a negative predictor for serum iron concentration [30, 31]. Interestingly, in our study, the odds of having severe anemia were higher in severely underweight women than those with average weight. Similarly, the odds of being anemic are higher among severely wasted children, which indicates the domination of nutritional deficiency anemia in the region [32, 33]. Women from younger age groups, such as adolescents, are more prone to being anemic due to increased requirement and decreased intake of iron, rapid physical growth, menstrual loss, and high iron demand for hemoglobin (Hgb) formation. Moreover, these women would slowly be adapting to the iron demand post‐menarche that might could have played a role [34].

## 5 Implications

Anemia contributes significantly to the morbidity and mortality of women in developing countries. Therefore, it is crucial to analyze the regional demographic data comprehensively and identify the closely linked factors that contribute to the development of anemia. The data and correlation are essential for designing effective healthcare policies and beneficiary schemes. Policymakers can formulate more targeted and impactful programs by identifying vulnerable populations and gaining a deeper understanding of the associated factors. Proper implementation and execution of these policies and programs can greatly benefit those affected by anemia, ultimately reducing the associated mortality and morbidity within the affected sub‐population. The data and healthcare models derived from these studies have the potential to provide substantial benefits not only to regional policymakers but also to those in other countries sharing similar demographic characteristics, especially within the developing world.

## 6 Limitations

This analysis relies on NFHS‐5 data, which may be updated in future NFHS‐6 datasets, potentially altering some findings. The focus on non‐pregnant females was necessary to maintain the study's scope but limits the generalizability of results to the broader population. The cross‐sectional nature of NFHS data restricts causal inferences and the ability to compare with other populations. Future studies should address these limitations by including a broader population and longitudinal data to enhance the robustness and generalizability of the findings.

## 7 Conclusions

This study reveals a marked divergence in the socio‐epidemiological correlates of anemia among non‐pregnant women in East and West UP, reflecting the broader demographic and contextual heterogeneity between the two regions. In West UP, higher odds of anemia were observed among women living in rural areas, those who are alcoholics (OR = 1.153; *p* = 0.000), those with heart disease (OR = 1.155; *p *< 0.01), younger women, those from economically disadvantaged backgrounds, illiterate women (OR = 1.335), and severely underweight women (OR = 1.523; *p* = 0.012). Conversely, in East UP, higher odds of anemia were associated with illiteracy (OR = 1.095; *p* = 0.010), having a child aged ≤48 months (OR = 1.296; *p* < 0.01), breastfeeding (OR = 1.067; *p* < 0.01), alcoholism (OR = 1.155; *p* < 0.01), hypertension (OR = 1.502; *p* < 0.01), and being thin (OR = 1.523; *p* = 0.012). These findings indicate that a one‐size‐fits‐all public health policy is insufficient. The data call for a paradigm shift toward precision public policy—an approach that aligns health interventions with the specific demographic, social, and clinical realities of each region. Such tailored strategies are essential to achieving more equitable and effective anemia control in India's diverse and stratified public health landscape.

## Author Contributions

**Sonam Maheshwari**: conceptualization, writing – original draft, project administration, software, formal analysis, data curation, methodology, validation, visualization. **Richa Sinha**: supervision, writing – original draft, methodology. **Neil Patel**: writing – original draft, investigation, methodology, formal analysis, writing – review and editing. **Okashah Kaiwan**: writing – original draft, formal analysis. **Avisham Goyal**: writing – original draft. **Oroshay Kaiwan**: writing – original draft, writing – review and editing, formal analysis, software, methodology, investigation. **Inderbir Padda**: writing – original draft, writing – review and editing. **Nidhi Uniyal**: writing – original draft, writing – review and editing, methodology, investigation, project administration, supervision. **Mohammed K. Suhail**: writing – original draft, writing – review and editing. **Talha Bin Emran**: writing – original draft, writing – review and editing, investigation, funding acquisition, project administration, formal analysis, software, data curation. **Nirja Kaka**: investigation, funding acquisition, writing – original draft, methodology, validation, visualization, writing – review and editing. **Yashendra Sethi**: conceptualization, investigation, writing – original draft, methodology, validation, visualization, writing – review and editing, software, formal analysis, project administration, supervision, resources.

## Ethics Statement

Data are available publicly, allowing the use of secondary data directly—hence, ethical approval was not required.

## Consent

NFHS is conducted with the informed consent of the population.

## Conflicts of Interest

The authors declare no conflicts of interest.

## Data Availability Stateme

NFHS‐5 data are available publicly.
